# Precise and Robust RTK-GNSS Positioning in Urban Environments with Dual-Antenna Configuration

**DOI:** 10.3390/s19163586

**Published:** 2019-08-17

**Authors:** Peirong Fan, Wenyi Li, Xiaowei Cui, Mingquan Lu

**Affiliations:** 1 Department of Electronic Engineering, Tsinghua University, Beijing 100084, China; 2 Satellite Navigation Center, Haidian District, Beijing 100094, China

**Keywords:** GNSS, RTK, centimeter-level positioning, dual-antenna reception, ADOP

## Abstract

Robust and centimeter-level Real-time Kinematic (RTK)-based Global Navigation Satellite System (GNSS) positioning is of paramount importance for emerging GNSS applications, such as drones and automobile systems. However, the performance of conventional single-rover RTK degrades greatly in urban environments due to signal blockage and strong multipath. The increasing use of multiple-antenna/rover configurations for attitude determination in the above precise positioning applications, just as well, allows more information involved to improve RTK positioning performance in urban areas. This paper proposes a dual-antenna constraint RTK algorithm, which combines GNSS measurements of both antennas by making use of the geometric constraint between them. By doing this, the reception diversity between two antennas can be taken advantage of to improve the availability and geometric distribution of GNSS satellites, and what is more, the redundant measurements from a second antenna help to weaken the multipath effect on the first antenna. Particularly, an Ambiguity Dilution of Precision (ADOP)-based analysis is carried out to explore the intrinsic model strength for ambiguity resolution (AR) with different kinds of constraints. Based on the results, a Dual-Antenna with baseline VEctor Constraint algorithm (RTK) is developed. The primary advantages of the reported method include: (1) Improved availability and success rate of RTK, even if neither of the two single-antenna receivers can successfully solve the AR problem; and (2) reduced computational burden by adopting the concept of measurement projection. Simulated and real data experiments are performed to demonstrate robustness and precision of the algorithm in GNSS-challenged environments.

## 1. Introduction

The recent interest in emerging Global Navigation Satellite System (GNSS) applications such as unmanned aerial vehicles (UAVs) and automatic driving systems has necessitated the development of robust and centimeter-level precise GNSS positioning. 

Real-time Kinematic (RTK) has been proven to be a reliable and efficient method for precise localization of outdoor vehicles in open areas [1]. In principle, integer ambiguity resolution (AR) is the key to RTK relative positioning. Traditional AR technique comprises two steps: Firstly, estimate the float ambiguities by solving the equations of GNSS measurements; and then, search for the correct integer ambiguities in ambiguity space with the center of the float ambiguities [2]. Therefore, precise estimation of float ambiguities, which are significantly influenced by satellite geometric distribution and the qualities of GNSS measurements, especially the ones of pseudoranges [3], is crucial for successful AR.

However, the performance of RTK deteriorates significantly in urban environments, since the number of visible satellites slumps because of frequent signal blockages in dynamic situations [1], and pseudorange measurements are contaminated with large multipath errors from trees and high-rises. As a result, the AR success rate declines dramatically and, therefore, RTK fails to provide accurate positioning results.

Intensive research efforts have been expanded to enhance GNSS precise positioning in harsh environments. The two mostly investigated directions are multi-sensor fusion [4,5,6] and robust GNSS positioning algorithms [7,8], respectively. Firstly, by integrating GNSS with self-contained sensors such as inertial navigation systems (INS), unmanned vehicles are able to maintain accurate navigation through GNSS signal outages for a short time, which is normally one minute or more [9]. Nevertheless, the challenge in GNSS/INS integrated positioning comes with its disability for long-term precise navigation during GNSS blockages due to the significant bias drift in consumer-grade INS modules, which are usually applied in commercial unmanned vehicles. Secondly, existing algorithms to improve the robustness of GNSS positioning against degraded environments mainly focus on the elimination of noisy GNSS measurements and anomalies, such as multipath detection algorithms [7] and Carrier phase-based Receiver Autonomous Integrity Monitoring (CRAIM) [8]. That is, the robustness and precision gains come at the cost of reduced availability. Therefore, in order to fulfil robust and low-cost precise positioning, a solution to enhance RTK availability and precision is still needed.

Fortunately, a new research direction appears, along with the use of multiple-antenna configuration in above-mentioned GNSS applications. The initial purpose of such configuration is to determine the attitude of the moving platform, which is denoted by orientation of the baselines between several onboard RTK antennas [4]. Nevertheless, raw GNSS observations from an auxiliary rover and the relationship between master and auxiliary rovers, in fact, could also be used to improve AR performance of the positioning system [10,11,12,13]. As according to Taro [14], pronounced measurement discrepancy between two antennas appears when vehicles maneuver through urban areas, since each GNSS antenna involves different GNSS signal propagation paths. Specifically, due to tree and building blockages, there is a high probability that rover antennas have different numbers of satellites in view, and even for common-viewed satellites, their measurement noise characteristics may differ from antenna to antenna. 

Previous studies on multi-antenna configured AR have focused on several recognized directions. One of the most popular research fields is high-reliability attitude determination [15]. Since the two antennas are closely placed, the common-mode errors can be removed almost completely by differencing their GNSS measurements, and therefore, orientation of the baseline is much easier to obtain. In dual-antenna attitude determination, a more efficient ambiguity-searching algorithm is the Multivariate Constraint Least-squares AMBiguity Decorrelation Adjustment (MC-LAMBDA) by Nadarajah [15,16]; while for low-cost GNSS receivers with ultrashort baseline, a Mean Square Residual (MSR) method was developed in [17]. Another research direction of multi-antenna reception is to improve the robustness of RTK-GNSS precise positioning. Firstly, at the fix solution level, where correct integer ambiguities have been obtained, Farhad [10] combined redundant GNSS fix solutions from three antennas with a weighted estimation method to increase precision of the positioning result. In addition, Taro [14] improved availability of the multi-antenna positioning outputs by transforming redundant antenna positions to the UAV master antenna position, using the previously solved vehicle attitude. Secondly, to improve the accuracy of the float ambiguity estimation, Paziewski [13] developed a Multiple Rover Constraints (MRC) algorithm to re-parameterize ambiguities of common-viewed satellites from auxiliary rover to phase center of the master rover. The MRC method reduced the positioning error of open-sky GNSS receivers from 2.9–2.7 cm, with a medium length baseline up to 70 km. However, in urban areas, applications of this method are significantly limited because of the reduced number of satellites visible from both antennas, as well as the enlarged noise in GNSS measurements. In summary, although these methods indeed increase precision and availability of RTK positioning by making use of the multi-antenna configuration under given environments, the AR success rate improvement at the float solution level is limited and therefore, there is still room for improvement.

The approach presented here aims at developing a method that takes full advantage of the measurement redundancy and diversity, along with the geometric constraints, between two closely placed rover antennas to improve accuracy of the float ambiguity estimation and, consequently, the AR success rate. In particular, to explore the instinct model strength for AR with different dual-antenna constraints, an innovative Ambiguity Dilution of Precision (ADOP)-based analysis is performed in this paper.

The contribution is organized as follows. In Section 2, a brief introduction of the fundamental model of dual-antenna constraint AR is firstly given, then gains from different constraint formulae are analyzed and compared, and thereafter, according to the results, a Dual-Antenna with baseline VEctor Constraint (DAVEC) algorithm is proposed. Section 3 demonstrates the robustness and availability of the proposed algorithm with both simulation and real GNSS data experimental results. At last, conclusions are drawn in Section 4.

## 2. Methodology

### 2.1. Functional Model

**Figure 1:**
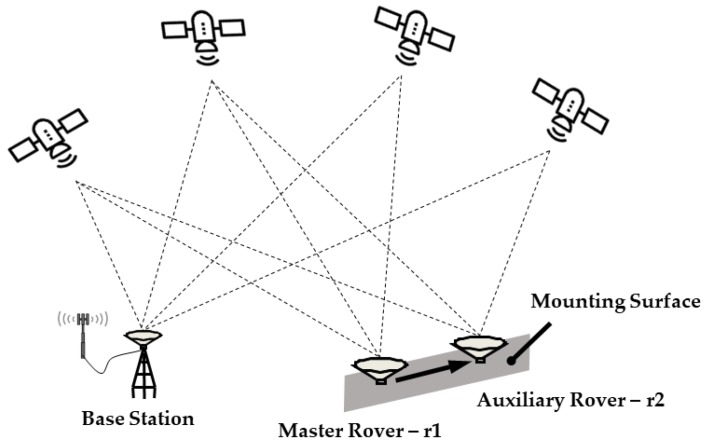
Dual-antenna configuration for precise Real-time Kinematic (RTK)-based Global Navigation Satellite System (GNSS) positioning.

Figure 1 schematically illustrates a dual-antenna configured RTK system. The base station is fixed in an open sky area and two GNSS rovers, termed as the master rover (r1) and the auxiliary rover (r2), respectively, are rigidly mounted on the surface of a vehicle. Positioning results of the system correspond to the phase center of the master rover. 

In terms of single-rover RTK, carrier phase and pseudorange observations are firstly differenced between satellites to remove receiver clock errors, and then, differenced again between rover and base receivers to remove common-mode errors, generating the following double-differenced (DD) observation model [18]:

(1) $$\begin{array}{cc} \lambda \nabla \Delta \Phi_{rb}^{ij} & =\nabla \Delta \rho_{rb}^{ij}+\lambda \nabla \Delta N_{rb}^{ij}+\nabla \Delta \varepsilon_{rb,\Phi }^{ij}\\ \nabla \Delta P_{rb}^{ij} & =\nabla \Delta \rho_{rb}^{ij}+\nabla \Delta \varepsilon_{rb,P}^{ij} \end{array}$$

where λ is the wavelength, Φ is the carrier phase observation in cycle, and P is the pseudorange measurement in meter. $\nabla \Delta {\left(\cdot \right)}_{rb}^{ij}=\Delta {\left(\cdot \right)}_{rb}^{i}-\Delta {\left(\cdot \right)}_{rb}^{j}$ with $\Delta {\left(\cdot \right)}_{rb}^{*}={\left(\cdot \right)}_{r}^{*}-{\left(\cdot \right)}_{b}^{*}$ is the double-differencing operator between base b and rover r with satellite i and j, ρ is the geometric range, N is the unknown carrier phase integer ambiguity, and ε represents all the errors that cannot be modeled. In this paper, satellite j is set as the reference satellite.

As mentioned before, traditional AR resolution of the observation model in Equation (1) uses a two-step procedure [11]. Firstly, estimate the float ambiguity solution by means of weighted least squares adjustment; secondly, search for the integer ambiguities with the well-known LAMBDA method. Once the AR problem is successfully solved, a centimeter-lever accurate baseline vector $r_{rb}$ can be obtained based on the resolved integer ambiguities, which is termed as the fixed solution.

However, in GNSS-challenged environments, the number of visible satellites from single-rover receivers may fail the minimum requirement for RTK positioning. And yet, for all that, it is worth noticing that the entire dual-antenna system may have adequate satellites in view altogether, such as the situation shown in Figure 1. What is more, as the two antennas are fixedly mounted on the vehicle, formulae about the ultrashort baseline between rovers actually impose several constraints that can be used to benefit the RTK precise positioning. Consequently, a dual-antenna constraint DD model is constructed:

(2) $$\begin{array}{l} \{\begin{array}{cc} \lambda \nabla \Delta \Phi_{r_{1}b}^{ij} & =\nabla \Delta \rho_{r_{1}b}^{ij}+\lambda \nabla \Delta N_{r_{1}b}^{ij}+\nabla \Delta \varepsilon_{r_{1}b,\Phi }^{ij}\\ \nabla \Delta P_{r_{1}b}^{ij} & =\nabla \Delta \rho_{r_{1}b}^{ij}+\nabla \Delta \varepsilon_{r_{1}b,P}^{ij} \end{array}\\ \{\begin{array}{cc} \lambda \nabla \Delta \Phi_{r_{2}b}^{pq} & =\nabla \Delta \rho_{r_{2}b}^{pq}+\lambda \nabla \Delta N_{r_{2}b}^{pq}+\nabla \Delta \varepsilon_{r_{2}b,\Phi }^{pq}\\ \nabla \Delta P_{r_{2}b}^{pq} & =\nabla \Delta \rho_{r_{2}b}^{pq}+\nabla \Delta \varepsilon_{r_{2}b,P}^{pq} \end{array}\\ y_{\xi }^{}=f\left(r_{r_{1}b}-r_{r_{2}b}\right) \end{array}$$

where $r_{1}$ and $r_{2}$ denote the master and the auxiliary rover, respectively, satellite j and q are reference satellites for each base and rover pair, and constraint function $f\left(r_{r_{1}b}-r_{r_{2}b}\right)$ is the known formula of the dual-antenna geometric constraint with prior information $y_{\xi }^{}$. 

Particularly, function $f\left(r_{r_{1}b}-r_{r_{2}b}\right)$ can be characterized as either a hard or a soft constraint. By definition, a hard constraint must be satisfied by any solution, while a soft constraint specifies a function to be optimized by choosing among the feasible solutions [19]. This paper takes $y_{\xi }^{}$ as a soft constraint to allow for measurement errors. Therefore, the measurement equation of the baseline constraint can be written as:

(3) $${\hat{y}}_{\xi }^{}=f\left(r_{r_{1}b}-r_{r_{2}b}\right)+\varepsilon_{\xi }^{}$$

where the “hat” symbol indicates measured quantities and $\varepsilon_{\xi }^{}$ is the error in ${\hat{y}}_{\xi }^{}$.

By linearizing Equations (2) and (3), a linearized dual-antenna constraint DD functional model is derived as follows:

(4) $$\left[\begin{array}{c} \lambda \nabla \Delta \Phi_{r_{1}b}^{}\\ \nabla \Delta P_{r_{1}b}^{}\\ \lambda \nabla \Delta \Phi_{r_{2}b}^{}\\ \nabla \Delta P_{r_{2}b}^{}\\ {\hat{y}}_{\xi }^{} \end{array}\right]=\left[\begin{array}{cccc} A_{n\times 3} & 0_{n\times 3} & \lambda I_{n\times n} & 0_{n\times m}\\ A_{n\times 3} & 0_{n\times 3} & 0_{n\times n} & 0_{n\times m}\\ 0_{m\times 3} & A_{m\times 3} & 0_{m\times n} & \lambda I_{m\times m}\\ 0_{m\times 3} & A_{m\times 3} & 0_{m\times n} & 0_{m\times m}\\ \Xi_{\xi }^{T} & -\Xi_{\xi }^{T} & 0_{p\times n} & 0_{p\times m} \end{array}\right]\left[\begin{array}{c} r_{r_{1}b}\\ r_{r_{2}b}\\ \nabla \Delta N_{r_{1}b}^{}\\ \nabla \Delta N_{r_{2}b}^{} \end{array}\right]+\left[\begin{array}{c} \nabla \Delta \varepsilon_{1,\Phi }^{}\\ \nabla \Delta \varepsilon_{1,P}^{}\\ \nabla \Delta \varepsilon_{2,\Phi }^{}\\ \nabla \Delta \varepsilon_{2,P}^{}\\ \varepsilon_{\xi }^{} \end{array}\right]$$

where n and m are number of unknown ambiguities of r1 and r2, respectively, matrix A includes the satellite/receiver geometry [9], ${Ι}_{n\times n}$ is an identity matrix of size n, and $\Xi_{\xi }^{}$ is the geometric matrix projecting vector $r_{r_{1}b}-r_{r_{2}b}$ to the constraint domain.

Accordingly, the measurement model above becomes an Integer Least Squares (ILS) problem with the form:

(5) $$y=Hx+\varepsilon ,x_{\nabla \Delta N}\in \mathbb{Z}$$

where H is the design matrix, x is the state vector, y is the measurement vector, and ε is the measurement noise with covariance matrix R.

### 2.2. ADOP Analysis for Three Types of Dual-Antenna Constraints

In this section, the specific formulae of different constraint function $f\left(r_{r_{1}b}-r_{r_{2}b}\right)$ are discussed.

As the two rover antennas are rigidly mounted on the surface of the platform, on the one hand, the geometric distance between antennas is fixed, while on the other hand, the baseline vector is relatively easy to obtain through the attitude determination. Therefore, three Dual-Antenna Constraint (DAC) strategies are evaluated in this paper, which are termed as: 

Strategy #1: Dual-Antenna with NO Constraint (DANOC). 

Strategy #2: Dual-Antenna with baseline LEngth Constraint (DALEC). The linearized constraint measurement equation is as follows:

(6) $${\hat{y}}_{\xi ,BL}^{}=\frac{{\hat{r}}_{r_{2}b}-{\hat{r}}_{r_{1}b}}{\left\|{\hat{r}}_{r_{2}b}-{\hat{r}}_{r_{1}b}\right\|}\left(r_{r_{2}b}-r_{r_{1}b}\right)+\varepsilon_{\xi ,BL}^{},\;\Xi_{\xi ,BL}=\frac{{\hat{r}}_{2}-{\hat{r}}_{1}}{\left\|{\hat{r}}_{2}-{\hat{r}}_{1}\right\|}$$

with baseline length noise variance of $R_{\xi ,BL}=\sigma_{\varepsilon }^{2}$, where ‖ ⋅ ‖ is the 2-norm operator for vectors.

Strategy #3: Dual-Antenna with baseline VEctor Constraint (DAVEC), and the corresponding measurement equation is derived as:

(7) $${\hat{y}}_{\xi ,BV}^{}=\left(r_{r_{2}b}-r_{r_{1}b}\right)+\varepsilon_{\xi ,BV}^{},\;\Xi_{\xi ,BV}=I_{3\times 3}^{}$$

with three-dimension baseline noise variance matrix of $R_{\xi ,BV}={\tilde{R}}_{\xi }^{}$.

Substituting Equations (6) and (7) into (4), we get the specific expression of strategies DALEC and DAVEC. To evaluate the performance gain of each strategy with respect to the single-antenna condition, the following two questions should be discussed:

1. Although new information is introduced to the single-rover observation model from both baseline constraints and GNSS measurements of the auxiliary rover, the number of states to be estimated increases too, making it harder to precisely resolve the float ambiguities. Then, is there any benefit from the dual-antenna combination?
2. As extra computation load is required to solve the expanded ILS measurement equations in the dual-antenna model, as well as to obtain the constraint observations in DALEC and DAVEC, we need to figure out which strategy should be chosen, and is there any method to reduce computational complexity of the algorithm?

In this paper, we take the AR success rate as an evaluating indicator, and specifically, introduce the Ambiguity Dilution of Precision (ADOP) to analyze the corresponding theoretical success rate.

#### 2.2.1. Ambiguity Dilution of Precision

The ADOP concept was introduced by Teunissen [20] as an easy-to-compute scalar diagnostic to evaluate the GNSS model strength for the AR problem. Despite its wide applications in analyzing single-rover baseline models [21], the ADOP has rarely been used in multi-antenna constraint AR analysis. The definition of ADOP is given as [20]:

(8) $$\mathrm{ADOP}={\sqrt{\left|Q_{\nabla \Delta \hat{N}}\right|}}^{\frac{1}{\nu }}$$

where ν is the number of unknown DD ambiguities, | ⋅ | is the determinant operator, and $Q_{\nabla \Delta \hat{N}}$ is the covariance matrix of float ambiguities. The structure of $Q_{\nabla \Delta \hat{N}}$ includes design and noise matrixes of the measurement model in Equation (4):

(9) $$Q={\left(H^{T}R^{-1}H\right)}^{-1}=\left[\begin{array}{cc} Q_{\hat{b}} & Q_{\hat{b}\nabla \Delta \hat{N}}\\ Q_{\hat{b}\nabla \Delta \hat{N}}^{T} & Q_{\nabla \Delta \hat{N}} \end{array}\right]$$

Accordingly, the AR success rate $P_{c}$ is bounded with:

(10) $$P_{c}\leq {\left[2\Phi \left(\frac{1}{2\mathrm{ADOP}}\right)-1\right]}^{\nu }$$

where Φ(x) denotes the cumulative distribution function of the standard normal distribution.

According to Equations (8) and (9), no additional measurements are required to compute the ADOP value. Furthermore, the geometric distribution of the satellites and measurement noise of GNSS observables—which as mentioned before, are the two key factors of successful AR—are denoted by the design matrix H and the noise matrix R, respectively.

#### 2.2.2. ADOP of the Dual-Antenna Constraint System

This section firstly deduces the general expression of Dual-Antenna Constraint ADOP, which is denoted by ${\mathrm{ADOP}}_{\mathrm{DAC}}$, and then discusses the specific forms of ${\mathrm{ADOP}}_{\mathrm{DAC}}$ under different strategies.

According to the ILS problem of (4), the observation model can be divided into two parts, one of which involves the joint DD measurements from r1 and r2, with related design matrix G, measurement noise covariance matrix$R_{G}$and measurement vector $y_{G}^{}$; the other involves the constraint with related design matrix $H_{\xi }^{}$, constraint noise covariance matrix $R_{\xi }$, and constraint vector $y_{\xi }^{}$. Therefore, linearized measurement equations in (4) can be rewritten as:

(11) $$\left[\begin{array}{c} {\hat{y}}_{G}^{}\\ {\hat{y}}_{\xi }^{} \end{array}\right]=\left[\begin{array}{c} G\\ H_{\xi }^{} \end{array}\right]x+\left[\begin{array}{c} \nabla \Delta \varepsilon \\ \varepsilon_{\xi }^{} \end{array}\right]$$

where ${\hat{y}}_{G}^{}={\left[\begin{array}{cccc} \lambda \nabla \Delta \Phi_{r_{1}b}^{T} & \nabla \Delta P_{r_{1}b}^{T} & \lambda \nabla \Delta \Phi_{r_{2}b}^{T} & \nabla \Delta P_{r_{2}b}^{T} \end{array}\right]}^{T}$ and $H_{\xi }^{}=\left[\begin{array}{ccc} \Xi_{\xi }^{T} & -\Xi_{\xi }^{T} & 0 \end{array}\right]$.

According to Equation (A1) in Appendix A, the dual-antenna ambiguity covariance matrix ${\tilde{Q}}_{\nabla \Delta \hat{N}}$ in Equation (8) is given as:

(12) $$\begin{array}{cc} {\tilde{Q}}_{\nabla \Delta \hat{N}} & ={\left(Q_{G}^{-1}+H_{\xi }^{T}R_{\xi }^{-1}H_{\xi }^{}\right)}^{-1}{}_{\nabla \Delta \hat{N}}\\ & ={\left(Q_{G}^{}-Q_{G}^{}H_{\xi }^{T}{\left(H_{\xi }^{}Q_{G}^{}H_{\xi }^{T}+R_{\xi }^{}\right)}_{}^{-1}H_{\xi }^{}Q_{G}^{}\right)}_{\nabla \Delta \hat{N}} \end{array}$$

where $Q_{G}^{}={\left(G^{T}R_{G}^{-1}G\right)}^{-1}$ is the covariance matrix of the constraint-free situation.

The relationship between $Q_{G}^{}$ and state covariance matrixes of r1 and r2 under single-antenna RTK conditions, which are represented by $Q_{1}^{}$ and $Q_{2}^{}$, respectively, satisfies:

(13) $$Q_{G}^{}=\left[\begin{array}{cccc} I_{3} & & & \\ & & I_{3} & \\ & I_{n} & & \\ & & & I_{m} \end{array}\right]\left[\begin{array}{cccc} Q_{b_{1}} & Q_{b_{1}\nabla \Delta N_{1}}^{} & & \\ Q_{b_{1}\nabla \Delta N_{1}}^{T} & Q_{\nabla \Delta N_{1}} & & \\ & & Q_{b_{2}} & Q_{b_{2}\nabla \Delta N_{2}}^{}\\ & & Q_{b_{2}\nabla \Delta N_{2}}^{T} & Q_{\nabla \Delta N_{2}} \end{array}\right]\left[\begin{array}{cccc} I_{3} & & & \\ & & I_{n} & \\ & I_{3} & & \\ & & & I_{m} \end{array}\right]$$

with

(14) $$\;Q_{1}=\left[\begin{array}{cc} Q_{b_{1}} & Q_{b_{1}\nabla \Delta N_{1}}^{}\\ Q_{b_{1}\nabla \Delta N_{1}}^{T} & Q_{\nabla \Delta N_{1}} \end{array}\right]\;,\;Q_{2}=\left[\begin{array}{cc} Q_{b_{2}} & Q_{b_{2}\nabla \Delta N_{2}}^{}\\ Q_{b_{2}\nabla \Delta N_{2}}^{T} & Q_{\nabla \Delta N_{2}} \end{array}\right]$$

Substituting $H_{\xi }^{}$ and Equation (13) into (12), we can get the joint covariance matrix ${\tilde{Q}}_{\nabla \Delta \hat{N}}$ and the corresponding ${\mathrm{ADOP}}_{\mathrm{DAC}}$:

(15) $${\mathrm{ADOP}}_{\mathrm{DAC}}={\sqrt{\left|\left[\begin{array}{cc} Q_{\nabla \Delta N_{1}}-Q_{b_{1}\nabla \Delta N_{1}}^{T}W_{\varepsilon }Q_{b_{1}\nabla \Delta N_{1}}^{} & Q_{b_{1}\nabla \Delta N_{1}}^{T}W_{\varepsilon }Q_{b_{2}\nabla \Delta N_{2}}^{}\\ Q_{b_{2}\nabla \Delta N_{2}}^{T}W_{\varepsilon }Q_{b_{1}\nabla \Delta N_{1}}^{} & Q_{\nabla \Delta N_{2}}-Q_{b_{2}\nabla \Delta N_{2}}^{T}W_{\varepsilon }Q_{b_{2}\nabla \Delta N_{2}}^{} \end{array}\right]\right|}}^{\frac{1}{n+m}}$$

where the weight matrix $W_{\varepsilon }$ can be expressed as:

(16) $$W_{\varepsilon }=\Xi_{\varepsilon }^{}{\left(\Xi_{\varepsilon }^{T}Q_{b_{1}}\Xi_{\varepsilon }^{}+\Xi_{\varepsilon }^{T}Q_{b_{2}}\Xi_{\varepsilon }^{}+R_{\varepsilon }^{}\right)}^{-1}\Xi_{\varepsilon }^{T}.$$

According to the equation above, $W_{\varepsilon }$ is essentially the inverse product of the joint positioning state error matrix with effects of the baseline measurement noise, as well as the single-antenna positioning state errors of r1 and r2 taken into consideration. To be specific, geometric covariance matrixes $Q_{b_{1}}$, $Q_{b_{2}}$, and $R_{\varepsilon }^{}$ are firstly superposed in the constraint domain and then transferred to the positioning domain by the geometry matrix Ξ.

Next, by substituting Equations (6), (7), and (16) into (15), the specific expressions of ADOP for the above three constraint strategies can be derived as follows:

(17) $${\mathrm{ADOP}}_{\mathrm{DANOC}}={\sqrt{\left|\left[\begin{array}{cc} Q_{\nabla \Delta N_{1}} & \\ & Q_{\nabla \Delta N_{2}} \end{array}\right]\right|}}^{\frac{1}{n+m}}={\left({\sqrt{\left|Q_{\nabla \Delta N_{1}}\right|}}^{\frac{1}{n}}\right)}^{\frac{n}{n+m}}{\left({\sqrt{\left|Q_{\nabla \Delta N_{2}}\right|}}^{\frac{1}{m}}\right)}^{\frac{m}{n+m}}$$

(18) $${\mathrm{ADOP}}_{\mathrm{DALEC}}={\sqrt{\left|\left[\begin{array}{cc} Q_{\nabla \Delta N_{1}} & \\ & Q_{\nabla \Delta N_{2}} \end{array}\right]-\left[\begin{array}{c} Q_{b_{1}\nabla \Delta N_{1}}^{T}\\ Q_{b_{2}\nabla \Delta N_{2}}^{T} \end{array}\right]W_{\varepsilon ,BL}\left[\begin{array}{cc} Q_{b_{1}\nabla \Delta N_{1}}^{} & Q_{b_{2}\nabla \Delta N_{2}}^{} \end{array}\right]\right|}}^{\frac{1}{n+m}}$$

(19) $${\mathrm{ADOP}}_{\mathrm{DAVEC}}={\sqrt{\left|\left[\begin{array}{cc} Q_{\nabla \Delta N_{1}} & \\ & Q_{\nabla \Delta N_{2}} \end{array}\right]-\left[\begin{array}{c} Q_{b_{1}\nabla \Delta N_{1}}^{T}\\ Q_{b_{2}\nabla \Delta N_{2}}^{T} \end{array}\right]W_{\varepsilon ,BV}\left[\begin{array}{cc} Q_{b_{1}\nabla \Delta N_{1}}^{} & Q_{b_{2}\nabla \Delta N_{2}}^{} \end{array}\right]\right|}}^{\frac{1}{n+m}}$$

where $W_{\varepsilon ,BV}={\left(Q_{b_{1}}+Q_{b_{2}}+R_{\varepsilon ,BV}^{}\right)}^{-1}$ and $W_{\varepsilon ,BL}=\frac{1}{\xi }\Xi_{\xi ,BL}\Xi_{\xi ,BL}^{T}$, with $\xi =\Xi_{\xi ,BL}^{}Q_{b_{1}}\Xi_{\xi ,BL}^{T}+\Xi_{\xi ,BL}^{}Q_{b_{2}}\Xi_{\xi ,BL}^{T}+R_{\xi ,BL}$. 

As can be seen, Equation (17) clearly shows that the ${\mathrm{ADOP}}_{\mathrm{DANOC}}$ is basically a weighted average of the single-antenna ADOP of r1 and r2. The weighting factor is assigned according to the number of unknown DD ambiguities; while in Equations (18) and (19), a correction term is added to the ambiguity covariance matrix of strategy DANOC. To be specific, the modification is theoretically a projection of the combined state errors of r1 and r2, which are represented by weighting matrix $W_{\varepsilon }$, from position domain to ambiguity domain. 

Moreover, noting that $W_{\varepsilon ,BV}$ is reversible, according to Appendix A, the formula of ${\mathrm{ADOP}}_{\mathrm{DAVEC}}$ can be transformed as follows:

(20) $${\mathrm{ADOP}}_{\mathrm{DAVEC}}{=\mathrm{ADOP}}_{\mathrm{DANOC}}{\sqrt{\frac{\left|\left(Q_{b_{1}}-Q_{b_{1}\nabla \Delta N_{1}}^{}Q_{\nabla \Delta N_{1}}^{-1}Q_{b_{1}\nabla \Delta N_{1}}^{T}\right)+\left(Q_{b_{2}}-Q_{b_{2}\nabla \Delta N_{2}}^{}Q_{\nabla \Delta N_{2}}^{-1}Q_{b_{2}\nabla \Delta N_{2}}^{T}\right)+R_{\varepsilon ,BV}^{}\right|}{\left|Q_{b_{1}}+Q_{b_{2}}+R_{\varepsilon ,BV}^{}\right|}}}^{\frac{1}{n+m}}$$

Apparently, compared with ${\mathrm{ADOP}}_{\mathrm{DANOC}}$, an extra combination gain in DAVEC from prior knowledge of the baseline vector turns up, which is represented by the second term on the right in Equation (20). In terms of physical meaning, in the numerator of the correction term above, the positioning state errors of both r1 and r2 are reduced, resulting in a smaller value than the dominator. However, since the weighting matrix $W_{\varepsilon ,BL}$ is irreversible, an explicit expression of the relationship between ${\mathrm{ADOP}}_{\mathrm{DANOC}}$ and ${\mathrm{ADOP}}_{\mathrm{DAVEC}}$ cannot be provided. 

#### 2.2.3. ADOP-Based Performance Gain Evaluation 

In order to examine the performance gain of above dual-antenna combination strategies, the ADOP-based instantaneous AR success rates are simulated and compared in the following scenarios: 

Scenario #0: Single-antenna (SANT) RTK as a reference;

Scenario #1: DANOC while each rover has one exclusive satellite that can only be observed by each antenna itself, and thus, the whole system has one more satellite in view than r1/r2;

Scenario #2-1: DALEC while two antennas share a common view of satellites;

Scenario #2-2: DALEC while each rover has one exclusive satellite;

Scenario #3-1: DAVEC while two antennas share a common view of satellites; and

Scenario #3-2: DAVEC while each rover has one exclusive satellite.

In all scenarios, receiver r1 and r2 observe an equal number of satellites ranging from 5 to 10 with a cut-off elevation angle of 15 degrees. Standard deviations (STDs) of the additive white Gaussian noise in r1 and r2 carrier phase and pseudorange measurements are 0.005 m and 0.5 m, respectively. STDs of the baseline length measurement in Scenarios 2-1 and 2-2 is 0.010 m, and STDs of the baseline vector measurements in Scenario 3-1 and 3-2 are 0.040 m and 0.080 m in horizontal and vertical directions, respectively. 

**Figure 2:**
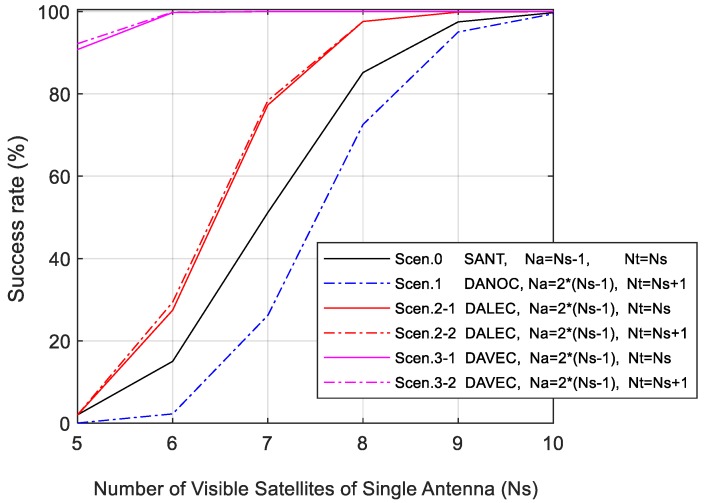
Simulated ambiguity resolution (AR) success rate of dual-antenna combinations with Dual-Antenna with NO Constraint (DANOC), Dual-Antenna with baseline LEngth Constraint (DALEC), and Dual-Antenna with baseline VEctor Constraint (DAVEC) compared with referencing single-antenna method.

Figure 2 shows the simulated AR success rate of above six scenarios. 

As depicted in the figure, the success rate increases as the number of visible satellites rises. However, compared with Scenario 0, a slump appears when two antennas are directly combined in Scenario 1. The reason is that, for DANOC, AR resolutions of r1 and r2 are mutually independent and the ADOP value for the master rover is equal to that in Scenario 0; however, the total number of unknown DD ambiguities of the dual-antenna system (Na) is doubled, thus the success rate drops to one-half of the root of the single-antenna AR. For DALEC, the AR estimation upper bound is increased by 10–20%; meanwhile, the emergence of exclusive satellites in Scenario 2-2 also brings some extra gain. The largest combination gain comes from DAVEC, whose AR success rate is greater than 90% for all simulated number of visible satellites.

According to the results above, no combination gain could be seen from DANOC, which means that the cost of increasing system unknowns is greater than the benefit of adding the information of rover r2. Nevertheless, with the help of prior constraint information of the baseline vector, DALEC and DAVEC can bring positive combination gains to the dual-antenna system. The best AR performance comes with the DAVEC method, and should be adopted in the dual-antenna algorithm design.

### 2.3. Precise Positioning with DAVEC

#### 2.3.1. Mechanism

In this section, a DAVEC-based RTK algorithm is developed. The basic idea of the algorithm is to generate a set of equivalent GNSS measurements of the master rover by projecting observations of the auxiliary rover to the phase center of the master rover with the use of the known baseline vector. 

The reason for the projection process is as follows. On the one hand, in terms of the ILS problem in Equation (4), the rover r2-related state parameters are of no concern to our system, but increase both computational burden and AR difficulty of the algorithm. On the other hand, with the early-solved baseline vector, no Degree of Freedom (DOF) remains between r1 and r2, meaning that the position and DD ambiguities of r2 can be determined uniquely by that of r1, along with the baseline vector. Therefore, with the procedure of projecting, a new dimension-reduced observation model can be obtained in the form of:

(21) $$\tilde{y}=f\left(\left[\begin{array}{c} r_{r_{1}b}\\ \nabla \Delta {\overset{⌣}{N}}_{r_{1}b}^{} \end{array}\right]\right)+\overset{⌣}{\varepsilon }$$

where $\nabla \Delta {\overset{⌣}{N}}_{r_{1}b}^{}$ is the equivalent DD ambiguities of all satellites in view of the dual-antenna system. Specifically, $\nabla \Delta {\overset{⌣}{N}}_{r_{1}b}^{}$ comprises of two parts: DD ambiguities of satellites observed by r1 and DD ambiguities of satellites observed by r2 only, denoted by $\nabla \Delta N_{r_{1}b}^{}$ and $\nabla \Delta {\tilde{N}}_{r_{2}b}^{}$, respectively.

Below, a four-step projection process for DAVEC-based RTK is introduced.

Step i: Select the reference satellites for rovers r1 and r2.

**Figure 3:**
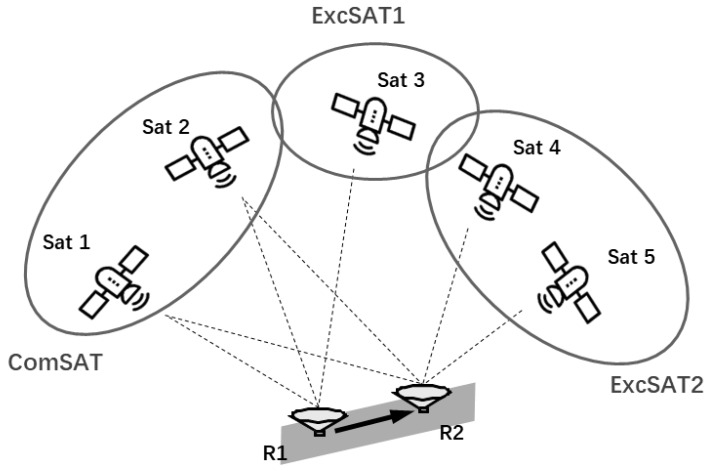
Classification of Line-of-Sight (LOS) satellites of the dual-antenna system according to each antenna’s visibility situation.

Line-of-Sight (LOS) satellites of the dual-antenna system can actually fall into three classes: Common-viewed ones observed by both r1 and r2, ones observed by r1 only, and ones observed by r2 only, which are named as classes *ComSAT*, *ExcSAT1,* and *ExcSAT2* in Figure 3, respectively.

Reference satellites for differencing between satellites are selected among the common visible satellites in class *ComSAT*. Specifically, if two or more satellites are observed by both r1 and r2, the one with the highest elevation angle (such as the Sat 2 in Figure 3) should be chosen as the reference satellite for both antennas. Otherwise, if the number of satellites in *ComSAT* is less than two, which means there are no common DD ambiguities between r1 and r2, the reference satellites are selected independently as the same as the single-antenna situation. 

Step ii: Project pseudorange observations of the auxiliary antenna to the phase center of the master rover.

The DD pseudorange measurement of rover r2 can be transferred to the phase center of r1 by adding a geometric range correction to it:

(22) $$\nabla \Delta {\tilde{P}}_{r_{1}b}^{pq}=\nabla \Delta P_{r_{2}b}^{pq}-\nabla \Delta {\hat{\rho }}_{r_{2}r_{1}}^{pq}$$

where q is the reference satellite of r2, p represents any other satellite except q, and $\nabla \Delta {\hat{\rho }}_{r_{2}r_{1}}^{pq}$ is the geometric range correction term, which can be approximated as:

(23) $$\nabla \Delta {\hat{\rho }}_{r_{2}r_{1}}^{pq}\approx -(1_{r_{2}}^{p}-1_{r_{2}}^{q})\cdot {\hat{r}}_{r_{2}r_{1}}$$

where ‘⋅’ denotes the dot operator, $1_{}^{p}$ is the LOS unit vector from the phase center of r2 to satellite p, and ${\hat{r}}_{r_{2}r_{1}}$ is the estimated baseline vector between r1 and r2.

Step iii: Project carrier phase observations of the auxiliary antenna to the phase center of the master rover.

In addition to geometric range corrections, the DD ambiguities between the DD carrier phases of r1 and r2 also need to be eliminated from the projection of DD carrier phase measurements of r2, since what we will estimate are the DD ambiguities of r1, not the ones of r2. Thus, the projection can be expressed as:

(24) $$\lambda \nabla \Delta {\tilde{\Phi }}_{rb_{1}}^{pq}=\lambda \nabla \Delta \Phi_{r_{2}b}^{pq}-\nabla \Delta {\hat{\rho }}_{r_{2}r_{1}}^{pq}-\lambda \nabla \Delta N_{r_{2}r_{1}}^{pq}$$

where $\nabla \Delta N_{r_{2}r_{1}}^{pq}$ is the DD ambiguity of the baseline vector $r_{r_{2}r_{1}}$. Specifically, since the measurement error in $\nabla \Delta \Phi_{r_{1}r_{2}}^{pq}$ is small, even in GNSS-challenged environments (within 0.25 cycle), and $\nabla \Delta {\hat{\rho }}_{r_{2}r_{1}}^{pq}$ is derived from the accurate baseline vector, the DD ambiguity $\nabla \Delta N_{r_{2}r_{1}}^{pq}$ can be determined as the rounding result of their difference, as shown in (25). One exception is that the involved satellite p or q is observed by r2 only and the DD ambiguities between r1 and r2 are inexistent; in this case, we set the corresponding DD ambiguities zero, which means we will solve the DD ambiguities of r2, instead.

(25) $$\nabla \Delta N_{r_{2}r_{1}}^{pq}=\{\begin{array}{l} \left[\nabla \Delta \Phi_{r_{2}r_{1}}^{pq}-\frac{\nabla \Delta {\hat{\rho }}_{r_{2}r_{1}}^{pq}}{\lambda }\right],\;\mathrm{if}\;\left(p,q\right)\;\mathrm{are}\;\mathrm{common}\;\mathrm{in}\;\mathrm{view}\\ 0,\;\mathrm{otherwise} \end{array}$$

where [⋅] is the rounding operator.

After the projection, DD ambiguities of r2 satellite pairs in class *ComSAT* are removed from the state vector in Equation (4). In other words, only DD ambiguities related to satellites in class *ExcSAT2* are added to the original single-antenna state vector, thus greatly reducing the computational burden, as well as the difficulty of solving the AR problem.

Step iv: Calculate covariance matrix of projected GNSS measurement noises of the auxiliary antenna.

To estimate noise characteristics of the projected measurements, we should take the baseline vector measurement noises into consideration. Namely, the projected measurement noise covariance matrix can be derived by superimposing the ranging noise in $\nabla \Delta \varepsilon_{2,\Phi ,P}^{}$ with that in $\nabla \Delta {\hat{\rho }}_{r_{2}r_{1}}^{pq}$.

(26) $${\tilde{R}}_{1,\Phi ,P}=R_{2,\Phi ,P}+D\Psi \cdot C_{n}^{e}R_{r_{2}r_{1}}^{n}{\left(C_{n}^{e}\right)}^{T}\Psi^{T}D^{T}$$

where $R_{2,\Phi ,P}$ is the noise covariance matrix of r2, $R_{r_{2}r_{1}}^{n}$ is the baseline vector noise matrix in the East-North-Up (ENU) coordinate, $C_{n}^{e}$ is the rotation matrix from ENU to the Earth-Center-Earth-Fixed (ECEF) coordinate, $\Psi ={\left[\begin{array}{cccc} 1_{r_{2}}^{1} & 1_{r_{2}}^{2} & \cdots & 1_{r_{2}}^{n} \end{array}\right]}^{T}$ is the geometric projection matrix, and D is the single-differencing matrix. 

In this paper, the ENU errors of ${\hat{r}}_{r_{2}r_{1}}$, which are denoted by $\sigma_{e}^{2}$, $\sigma_{n}^{2}\;$, and $\sigma_{u}^{2}$ are mutually independent, thus $R_{r_{2}r_{1}}^{n}$ can be formulated as a diagonal matrix with $R_{r_{2}r_{1}}^{n}=diag\left(\begin{array}{ccc} \sigma_{e}^{2} & \sigma_{n}^{2} & \sigma_{u}^{2} \end{array}\right)$. In practice, the baseline vector noise matrix can also be obtained during the attitude determination of the baseline vector. A detailed derivation of Equations (22)–(26) is provided in Appendix B. 

Consequently, the joint covariance matrix of measurements $\nabla \Delta {\tilde{P}}_{r_{1}b}^{pq}$ and $\lambda \nabla \Delta {\tilde{\Phi }}_{rb_{1}}^{pq}$ is expressed as:

(27) $${\tilde{R}}_{1}=\left[\begin{array}{cc} {\tilde{R}}_{1,\Phi } & 0\\ 0 & {\tilde{R}}_{1,P} \end{array}\right]$$

#### 2.3.2. Functional Model of DAVEC-Based RTK

Based on Equations (22)–(27), a dimension-reduced DAVEC measurement model is re-constructed as follows:

(28) $$\begin{array}{cc} \lambda \nabla \Delta \Phi_{r_{1}b}^{ij} & =\nabla \Delta \rho_{r_{1}b}^{ij}+\lambda \nabla \Delta N_{r_{1}b}^{ij}+\nabla \Delta \varepsilon_{r_{1}b,\Phi }^{ij}\\ \nabla \Delta P_{r_{1}b}^{ij} & =\nabla \Delta \rho_{r_{1}b}^{ij}+\nabla \Delta \varepsilon_{r_{1}b,P}^{ij}\\ \lambda \nabla \Delta {\tilde{\Phi }}_{r_{1}b}^{iq} & =\nabla \Delta \rho_{r_{1}b}^{iq}+\lambda \nabla \Delta N_{r_{1}b}^{iq}+\nabla \Delta {\tilde{\varepsilon }}_{r_{1}b,\Phi }^{iq}\\ \nabla \Delta {\tilde{P}}_{r_{1}b}^{iq} & =\nabla \Delta \rho_{r_{1}b}^{iq}+\nabla \Delta {\tilde{\varepsilon }}_{r_{1}b,P}^{iq}\\ \lambda \nabla \Delta {\tilde{\Phi }}_{r_{1}b}^{pq} & =\nabla \Delta \rho_{r_{1}b}^{pq}+\lambda \nabla \Delta N_{r_{2}b}^{pq}+\nabla \Delta {\tilde{\varepsilon }}_{r_{1}b,\Phi }^{pq}\\ \nabla \Delta {\tilde{P}}_{r_{1}b}^{pq} & =\nabla \Delta \rho_{r_{1}b}^{pq}+\nabla \Delta {\tilde{\varepsilon }}_{r_{1}b,P}^{pq} \end{array}$$

where the third and fourth equations in (28) correspond to r2-visible satellites in class *ComSAT*, and the fifth and sixth ones correspond to those in class *ExcSAT2*.

After linearizing the original and projected DD measurements with respect to the state vector in Equation (21), a new linearized DAVEC model reads as:

(29) $$\underset{y}{\underset{︸}{\left[\begin{array}{c} \lambda \nabla \Delta \Phi_{r_{n1}b}^{}\\ \lambda \nabla \Delta \Phi_{r_{n2}b}^{}\\ \nabla \Delta P_{r_{n1}b}^{}\\ \nabla \Delta P_{r_{n2}b}^{}\\ \lambda \nabla \Delta {\tilde{\Phi }}_{r_{m1}b}^{}\\ \lambda \nabla \Delta {\tilde{\Phi }}_{r_{m2}b}^{}\\ \nabla \Delta {\tilde{P}}_{r_{m1}b}^{}\\ \nabla \Delta {\tilde{P}}_{r_{m2}b}^{} \end{array}\right]}}=\underset{H}{\underset{︸}{\left[\begin{array}{cccc} A_{n_{1}\times 3} & \lambda I_{n_{1}\times n_{1}} & 0_{n_{1}\times n_{2}} & 0_{n_{1}\times m_{2}}\\ A_{n_{2}\times 3} & 0_{n_{2}\times n_{1}} & \lambda I_{n_{2}\times n_{2}} & 0_{n_{2}\times m_{2}}\\ A_{n_{1}\times 3} & 0_{n_{1}\times n_{1}} & 0_{n_{1}\times n_{2}} & 0_{n_{1}\times m_{2}}\\ A_{n_{2}\times 3} & 0_{n_{2}\times n_{1}} & 0_{n_{2}\times n_{2}} & 0_{n_{2}\times m_{2}}\\ A_{m_{1}\times 3} & 0_{m_{1}\times n_{1}} & \lambda I_{m_{1}\times n_{2}} & 0_{m_{1}\times m_{2}}\\ A_{m_{2}\times 3} & 0_{m_{2}\times n_{1}} & 0_{m_{2}\times n_{2}} & \lambda I_{m_{2}\times m_{2}}\\ A_{m_{1}\times 3} & 0_{m_{1}\times n_{1}} & 0_{m_{1}\times n_{2}} & 0_{m_{1}\times m_{2}}\\ A_{m_{2}\times 3} & 0_{m_{2}\times n_{1}} & 0_{m_{2}\times n_{2}} & 0_{m_{2}\times m_{2}} \end{array}\right]}}\underset{\tilde{x}}{\underset{︸}{\left[\begin{array}{c} r_{r_{1}b}\\ \nabla \Delta N_{r_{n1}b}^{}\\ \nabla \Delta N_{r_{n2}b}^{}\\ \nabla \Delta N_{r_{m2}b}^{} \end{array}\right]}}+\underset{\varepsilon }{\underset{︸}{\left[\begin{array}{c} \nabla \Delta \varepsilon_{r_{n1}b,\Phi }^{}\\ \nabla \Delta \varepsilon_{r_{n2}b,\Phi }^{}\\ \nabla \Delta \varepsilon_{r_{n1}b,P}^{}\\ \nabla \Delta \varepsilon_{r_{n2}b,P}^{}\\ \lambda \nabla \Delta {\tilde{\varepsilon }}_{r_{m1}b,\Phi }^{}\\ \lambda \nabla \Delta {\tilde{\varepsilon }}_{r_{m2}b,\Phi }^{}\\ \nabla \Delta {\tilde{\varepsilon }}_{r_{m1}b,P}^{}\\ \nabla \Delta {\tilde{\varepsilon }}_{r_{m2}b,P}^{} \end{array}\right]}}$$

where $n_{2}=m_{1}$ is the number of satellites in class *ComSAT*, $n_{1}$ and $m_{2}$ are satellites visible only in r1 and r2, respectively. As can be seen, positioning state parameters $r_{r_{2}b}$ and the unknown DD ambiguities of r2, which can be calculated from those of r1, are eliminated from the original functional model in Equation (4), so are the constraint measurement equations. 

Finally, after solving the ILS problem in Equation (29), a more accurate float solution can be obtained, thus facilitating the subsequent AR search.

Here, a simple discussion is given below to provide a qualitative explanation for the performance gain from DAVEC. For simplicity, considering a single GNSS system and single-frequency dual-antenna system, we denote s and t as the number of visible satellites in r1 and r2, respectively, l as the number of total DD measurements, ω as the number of system states, and k as the number of satellites observed by the auxiliary antenna only. The formulae of l and ω are given as:

(30) $$\begin{array}{l} l=2\left(s-1\right)+2\left(t-1\right)\\ \omega_{DANOC}=6+(s-1)+\left(t-1\right)\\ \omega_{DAVEC}=3+\left(s-1\right)+k \end{array}$$

Taking s=t=4,6,8 and k=0,1 for example, the number of measurements and unknowns of DANOC and DAVEC are compared in Table 1. 

As can be seen, compared with DANOC, the number of unknowns is reduced by half in DAVEC with *k* = 0 and is as the same as the one in the single-rover situation. In this sense, the performance gain is obtained from redundant observations of the same satellite. Moreover, in the condition of *k* = 1, although one more DD ambiguity is introduced, an improved satellite distribution is provided, which becomes another source of the DAVEC performance gain. Therefore, the float solution is enhanced by two factors: Redundancy in GNSS observations and improvement of the satellite geometric distribution.

## 3. Experiments

In this section, simulation experiments are firstly conducted to analyze the effects of measurement noises on AR performance of DAVEC, then road tests in urban areas are carried out for performance validation of the algorithm. 

### 3.1. ADOP-Based AR Success Rate Simulation 

**Figure 4:**
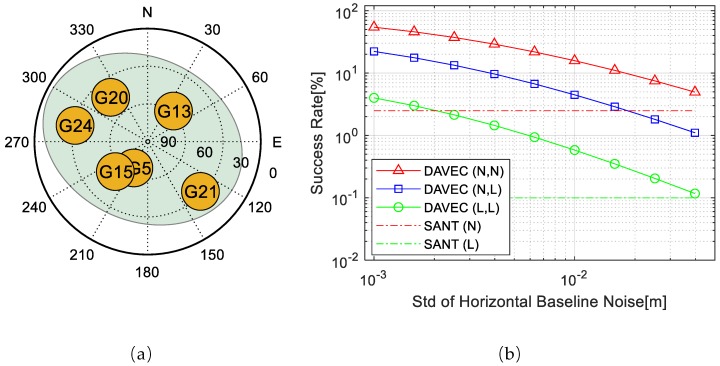
(**a**) Satellite geometry distribution. (**b**) Ambiguity Dilution of Precision (ADOP)-based AR Success rate of DAVEC and Single Antenna (SANT) algorithms under (**a**).

To illustrate the effects of different factors on the DAVEC algorithm, an ADOP-based success rate simulation is conducted, as shown in Figure 4b, with normal/large measurement noises of r1 and r2, and different baseline vector estimation noises. Code pseudorange and carrier phase noise STDs of the large noise situation are 1.0 m and 0.052 cycle, respectively, and the ones of the normal noise situation are 0.5 m and 0.026 cycle, respectively. Simulation experiments are designed with both antennas that suffer from large measurement noises (L, L), both have normal noises (N, N), and the noise STDs of r2 are significantly greater than that of r1 (N, L). Baseline vector noise STDs in horizontal range from 0.001 to 0.05 m, and in vertical the values are doubled, under the assumption that the DD ambiguities between r1 and r2 are resolved correctly. The AR success rate of single-antenna RTK with r1 is used as a reference. The satellite distribution is plotted in Figure 4a, with six visible satellites in a shared view of r1 and r2.

As can be seen, the success rate drops as the baseline noise rises, and when the noise exceeds a certain threshold, the AR success rate of DAVEC becomes lower than that of SANT. For noise combinations (L, L) and (N, N), the thresholds of noise STDs in horizontal are greater than 0.05 m, which is approximately the upper bound value for correct AR estimations between r1 and r2. While for the combination with r2 GNSS measurements much more deteriorated than those of r1, a dual-antenna combination gain still exists when the horizontal noise STD is smaller than 0.016 m. 

**Figure 5:**
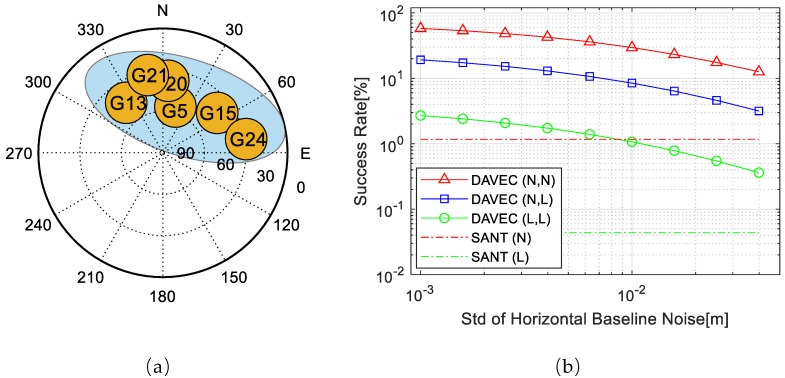
(**a**) Satellite geometry distribution. (**b**) ADOP-based AR Success rate of DAVEC and SANT algorithms under (**a**).

In addition, a similar simulation under a poor satellite distribution is conducted with all the six visible satellites distributed in the northern sky. The success rate and the satellite geometry distribution are plotted in Figure 5. With such satellite geometry distribution, the success rate drops greatly in comparison with the situation under normal geometry distribution. For all measurement noise combinations (L,L), (N,N), and (N,L), the proposed DAVEC method always has performance advantages over SANT over the range of simulated baseline noises. Here, comparing with the simulation experiment above, under normal satellite distributions, a larger baseline noise STD range is seen due to the significantly decreased referencing single antenna AR success rate.

In fact, the ENU error STDs of baseline vectors in urban environments are usually at the level of 0.005 m in horizontal and of 0.010 m in vertical. Meanwhile, in most GNSS dual-antenna applications, the distance between the two antennas is less than 1.5 m [22]. 

### 3.2. Road Test 

#### 3.2.1. Data Collection

**Figure 6:**
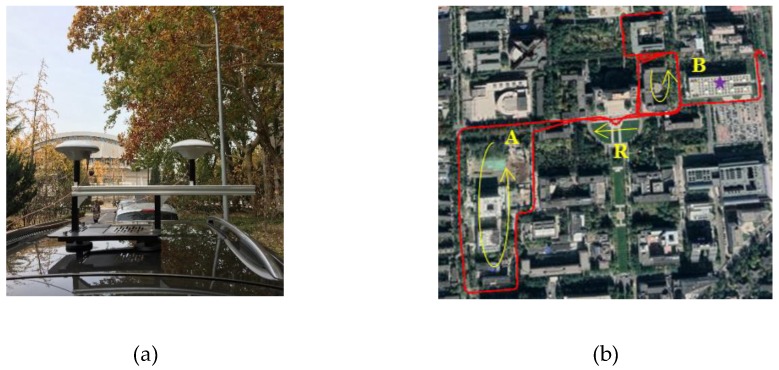
(**a**) Experimental platform configured with two GNSS antennas for raw observation data collection. (**b**) Road test routine in Tsinghua University, Beijing, China, including three dynamic scenarios through dense foliage, urban canyons, and open areas, represented by characters A, B, and R, respectively. The RTK base station was located at the position marked with a star.

Figure 6 shows the dual-antenna experiment platform and the road test routine, respectively. Two Harxon GPS500 GNSS antennas were installed on the top of a vehicle with a distance of 40 cm, and the Trimble MB-Two dual-frequency GNSS receiver was employed for GPS L1CA raw measurements. The base station for RTK was located on the rooftop of the Weiqing building. Test scenarios include dense foliage, urban canyons, and open areas, with a driving speed of around 20 km/h.

**Figure 7:**
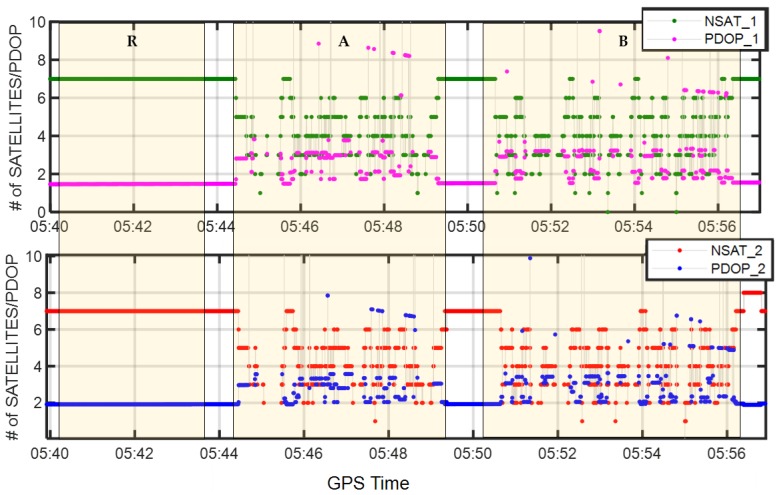
Number of visible satellites and values of Position Dilution of Precision (PDOP) in different scenarios for the master rover r1 (**top**) and the auxiliary rover r2 (**bottom**).

Figure 7 depicts the number of satellites visible in the master and auxiliary antennas during the whole routine, along with the Position Dilution of Precision (PDOP) parameter, which is a common indicator of the effect of satellite geometric distributions on positioning. Theoretically, the smaller the PDOP is, the more precise the positioning results will be. 

As can be seen, in the open area, up to seven satellites were visible, and the PDOPs were 1.5 and 1.9 for r1 and r2, respectively. While in GNSS-challenged environments, the number of satellites in view fluctuated significantly from zero to seven, and the PDOPs reached at as much as 8.0. Strong PDOP values (>5.0) appeared occasionally in test scenarios A and B because of a quite small number of visible satellites (only four at most times) and the poor satellite geometric distribution. However, it is worth noting that, although individual rovers suffered from insufficient satellites, a complementary behavior appeared between two rovers with different numbers of visible satellites and different satellites distributions. This reception diversity could bring benefits for RTK precise positioning in urban environments by dual-antenna combination. 

#### 3.2.2. Dual-Antenna ADOPs 

**Figure 8:**
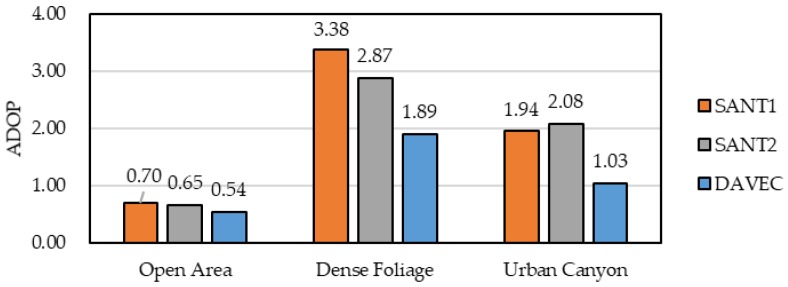
The average ADOPs of the master rover (SANT1), auxiliary rover (SANT2), and DAVEC in open areas and GNSS-obstructed observing environments.

Figure 8 compares the average ADOPs of the master rover (SANT1), the auxiliary rover (SANT2), and the DAVEC configuration with both antennas. It can be seen that, on the one hand, for both single and dual-antenna RTK algorithms, the open area environment gave the smallest ADOPs, while driving through dense foliage (Scenario #A) provided the largest ADOPs. On the other hand, compared with the master rover, a decline of ADOP appeared in DAVEC, with a percentage of 44.2% and 47.0% in Scenarios #A and #B, respectively. As mentioned before, this ADOP gain comes from the geometric distribution improvement and measurement redundancy.

#### 3.3.3. Positioning Results

In this paper, single-epoch RTK positioning results are calculated to facilitate performance demonstration of the proposed DAVEC algorithm.

**Figure 9:**
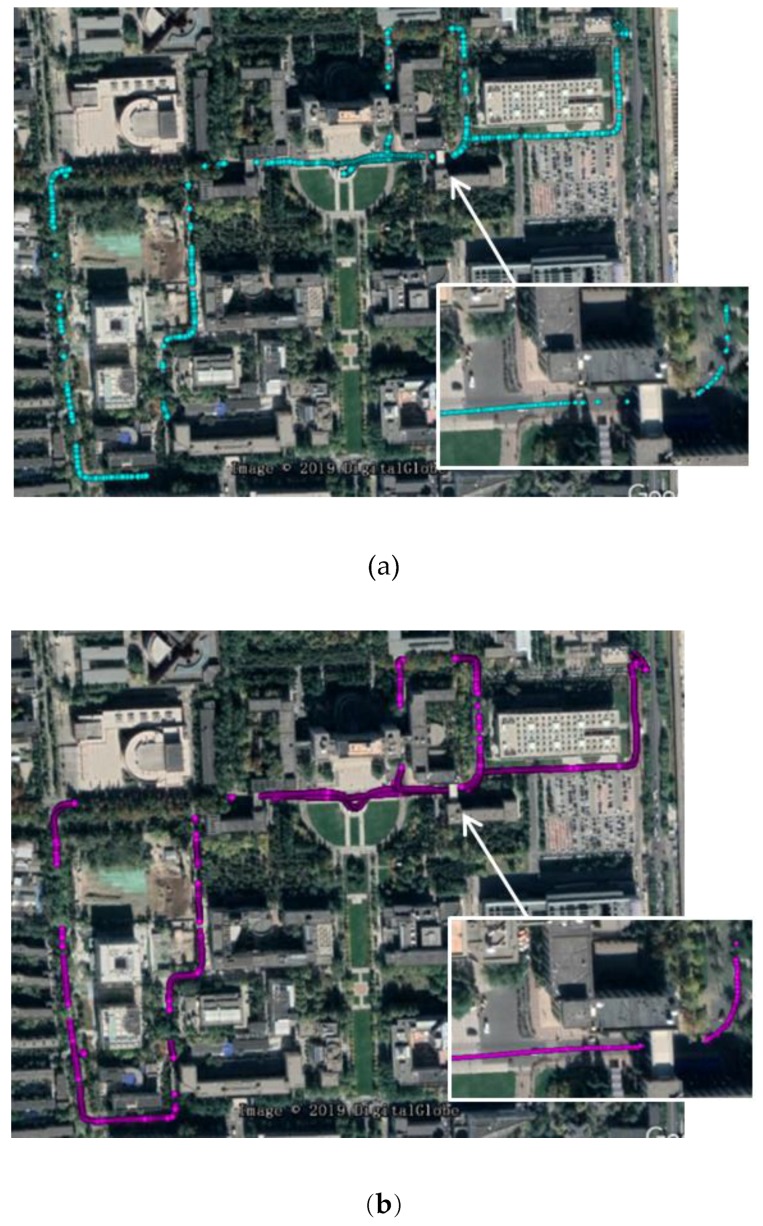
Single-epoch precise positioning results of: (**a**) SANT1 and (**b**) DAVEC.

Figure 9 depicts precise positioning results of the single rover and DAVEC–based RTK, as well as a partial enlargement near the skywalk between buildings. As can be seen, the proposed algorithm recovered more quickly from GNSS blockages and provided more robust RTK positioning solutions. According to statistic results, the RTK success rate is improved from 48.4% to 85.3% for the entire test routine. The success rate is defined as the proportion of fixed AR results to all the float results.

Moreover, at each epoch, the auxiliary antenna r2 either observes the same satellites as r1, or has one or more different satellites from r1, which are termed as the “Identical View” situation and “Non-identical View” situation, respectively. Namely, in the first case, improvement of AR performance of the DAVEC algorithm only comes from the GNSS measurement redundancy, while in the second case, satellite geometric distribution is also improved. 

To explore the contribution of the above two factors in the performance gain of DAVEC-based RTK, the number of AR fixed positioning results under different satellite visibility conditions of r1 are counted in Table 2. For each number of r1-observed satellites, statistics on the number of Identical View and Non-identical View situations are given, as well as the percentage of availability improvement for DAVEC compared with SANT1.

It can be seen from Table 2 that, when the master antenna fails to observe sufficient GNSS satellites (Nsat ≤ 3), the proposed algorithm may successfully solve the ambiguities by the procedure of dual-antenna combination. When the number of r1-visible satellites ranges from four to six, significant AR performance gain is obtained for both Identical View and Non-identical View situations. As last, when the Nsat reaches seven, the DAVEC algorithm can still improve the AR success rate moderately, even though the single antenna RTK can already achieve a relative high AR success rate.

In addition, even though the relative improvement percentage is high, the absolute number of AR fixed epochs enhancement was from 4382/16,683 (26.3%) to 10,541/16,683 (63.2%), meaning that there is still room for improvement in future applications of the DAVEC algorithm. Particularly, if an Inertial Measurement Unit module can be introduced in for attitude determination of the on-board baseline vector, a higher AR success rate can be expected.

Hereafter, precision and availability of the DAVEC-based RTK positioning results in different GNSS signal environments will be discussed.

i. Open Sky Environment Test

**Figure 10:**
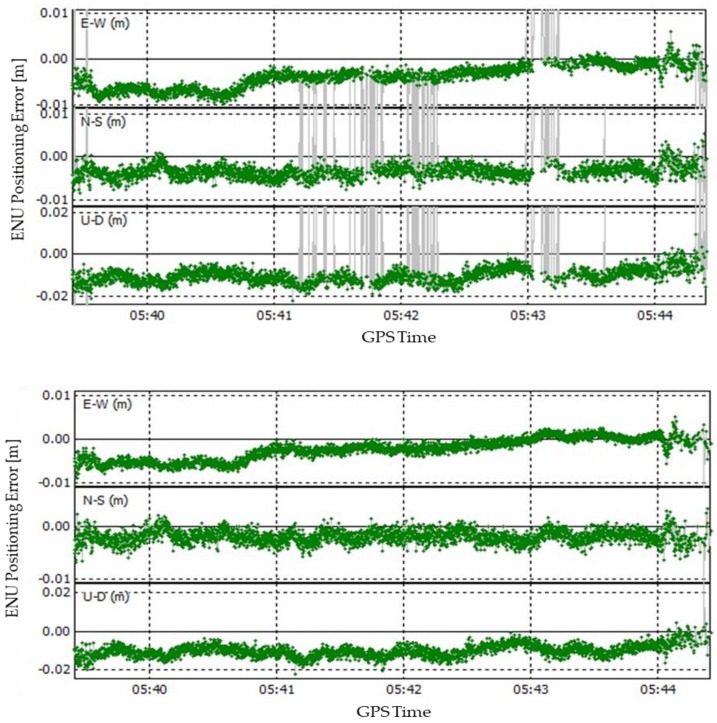
East-North-Up (ENU) Positioning errors of single rover RTK (**top**) and DAVEC-based RTK (**bottom**) algorithms in open areas from GPS time 5:39:25 to 5:44:25.

Figure 10 illustrates the positioning errors of single-rover and DAVEC-based RTK in the ENU coordinate, respectively. The reference trajectory is obtained from a self-developed dual-frequency (GPS L1C/A+L2) high-precision post processing GNSS software receiver. It is evident from the graph that many outliers in SANT1 positioning results appear with incorrect integer ambiguities due to limited number of visible satellites, which is six in the test environment. Nevertheless, almost no abnormal values have been found in DAVEC-based positioning results. The three-dimension STDs are determined on the basis of the shown errors, which for SANT1 are 0.022 m, 0.061 m, and 0.197 m, respectively, and for DAVEC are 0.003 m, 0.005 m, and 0.020 m, respectively.

ii. Urban Environment Test

**Figure 11:**
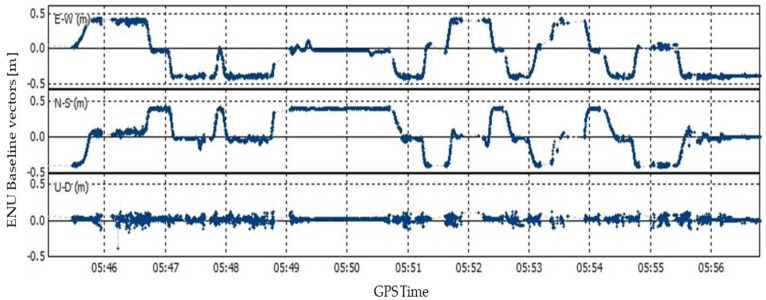
Dual-antenna baseline measurements for dynamic experiments under urban environments. (Scenario #A: From GPS time 5:44:30 to 5:49:00; Scenario #B: From GPS time 5:50:00 to 5:57:00.)

Figure 11 shows the estimated baseline vector between r1 and r2 for dynamic experiments under urban environments in the ENU coordinate. The MSR method in [17] was adopted, since it is suitable for single-epoch attitude estimation of ultra-short baselines from 0.2 to 1.5 m in length. Statistic results show that the success rate of attitude determination for Scenarios #A and #B are 90.6% and 83.5%, respectively. In UAVs and other GNSS dual-antenna applications, an INS module is usually configured and can be used to enhance attitude determination of the platform.

**Figure 12:**
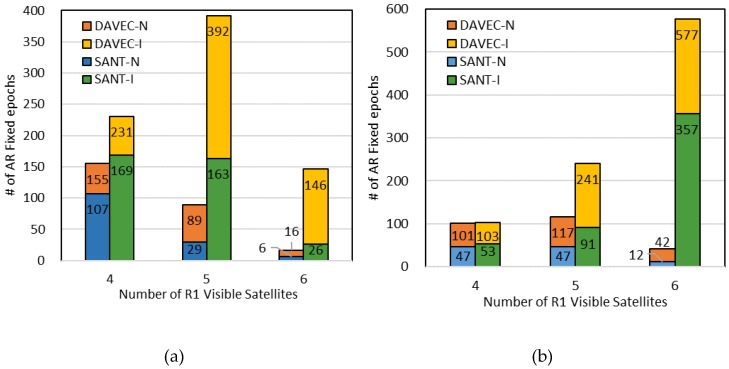
Statistics for AR-succeeded epochs with DAVEC and single rover RTK with respect to the number of visible satellites from the master rover for: (**a**) Scenario #A and (**b**) Scenario #B.

Figure 12 gives the RTK positioning results under different satellite availability conditions of the master antenna during test Scenarios #A and #B. In Figure 12a, the total number of AR fixed epochs is 500 for SANT1 and 1108 for the DAVEC algorithm with an increase of 123.6%. For most epochs, the number of satellites observed by r1 is four or five, and the identical view satellite condition is more likely to appear than the non-identical view one. While in Figure 12b, the total number of AR fixed epochs is 607 for SANT1 and 1221 for the DAVEC algorithm with an increase of 101.2%. Since in building-blocked environments, the satellite is only obstructed occasionally and from different antennas, the non-identical view condition has a relatively higher probability to occur. To sum up, in test environments with dense foliage and urban canyons, the characteristics of satellite reception diversity between two antennas are different, but in both situations, a great AR performance gain from the proposed algorithm can be seen. 

## 4. Conclusions

To realize robust and precise RTK-GNSS positioning with a dual-antenna-configured system in GNSS-challenged environments, this paper proposed a Dual-Antenna RTK algorithm with baseline VEctor Constraint, namely the DAVEC. By comparing the model strength of DAVEC with DANOC and DALEC through an innovative dual-antenna ADOP method, performance gain of DAVEC was illustrated. A less computational DAVEC-RTK algorithm was proposed based on the concept of measurement projection, in which the reception diversity between two antennas are taken advantage of to improve satellite distribution of the master rover. Experiment results show that in the given test environment, the system AR success rate has been improved from 48.4% to 85.3%, and the three-dimension precision of the relative positioning under open sky condition is within 0.02 m. Therefore, the algorithm is able to provide more precise positioning results with higher RTK success rate.

This paper discussed the algorithm with two antennas, but it can easily be expanded to situations with more antennas. The integration of DAVEC-based RTK with self-contained sensors, such as the INS, will be further researched in our future works.

## Figures and Tables

**Table 1:** Number of GNSS measurements and unknowns of DANOC and DAVEC.

s/t	l	ω		
		DANOC	DAVEC, *k* = 0	DAVEC, *k* = 1
4	12	12	6	7
6	20	16	8	9
8	28	20	10	11

**Table 2:** Number of AR fixed epochs with different satellite visibility with respect to non-identical and identical viewed satellite conditions.

Nsat (r1)	SANT1			DAVEC			IMPR
	Non	Iden	Total	Non	Iden	Total	
≤3	-	-	-	355	-	355	-
4	335	329	664	543	481	1024	54.2%
5	294	748	1042	1216	1876	3092	196.7%
6	121	1568	1689	498	4461	4959	193.6%
7	-	987	987	-	1111	1111	12.6%
Total	750	3632	4382	2612	7929	10,541	140.6%

## Appendix A

### Appendix A.1. Mathematical Formulae Used in the Dual-Antenna ADOP Derivation

The following formulae are used in the derivation of the dual-antenna constraint ADOPs.

If matrixes A and C are reversible,

(A1) $${\left(A+BCD\right)}^{-1}=A^{-1}-A^{-1}B{\left(DA^{-1}B+C^{-1}\right)}^{-1}DA^{-1}$$

If $A=\left[\begin{array}{cc} A_{11} & A_{12}\\ A_{21} & A_{22} \end{array}\right]$, we have:

(A2) $$A^{-1}=\left[\begin{array}{cc} {\left(A_{11}-A_{12}A_{22}^{-1}A_{21}\right)}^{-1} & -{\left(A_{11}-A_{12}A_{22}^{-1}A_{21}\right)}^{-1}A_{12}A_{22}^{-1}\\ -{\left(A_{22}-A_{21}A_{11}^{-1}A_{12}\right)}^{-1}A_{21}A_{11}^{-1} & {\left(A_{22}-A_{21}A_{11}^{-1}A_{12}\right)}^{-1} \end{array}\right]$$

and,

(A3) $$\begin{array}{cc} \det\left(A\right) & =\det\left(A_{22}\right)\det\left(A_{11}-A_{12}A_{22}^{-1}A_{21}\right)\\ & =\det\left(A_{11}\right)\det\left(A_{22}-A_{21}A_{11}^{-1}A_{12}\right) \end{array}$$

If matrixes A and B are reversible:

(A4) $$\left|A-DB^{-1}C\right|\left|B\right|=\left|A\right|\left|B-CA^{-1}D\right|$$

### Appendix A.2. Derivation of the ADOP with Baseline Vector Constraint

Hereafter, the derivation of Equation (20) is described.

For a DANOC-based dual-antenna combination, the covariance matrix ${\tilde{Q}}_{\nabla \Delta \hat{N}}$ is given as:

(A5) $${\tilde{Q}}_{\nabla \Delta \hat{N}}=\left[\begin{array}{cc} Q_{1} & \\ & Q_{2} \end{array}\right]-\left[\begin{array}{c} Q_{{\hat{b}}_{1}}\\ Q_{{\hat{b}}_{1}\nabla \Delta {\hat{N}}_{1}}^{T}\\ -Q_{{\hat{b}}_{2}}\\ -Q_{{\hat{b}}_{2}\nabla \Delta {\hat{N}}_{2}}^{T} \end{array}\right]\left[\begin{array}{cccc} Q_{{\hat{b}}_{1}} & Q_{{\hat{b}}_{1}\nabla \Delta {\hat{N}}_{1}}^{} & -Q_{{\hat{b}}_{2}} & -Q_{{\hat{b}}_{2}\nabla \Delta {\hat{N}}_{2}}^{T} \end{array}\right]$$

Substituting the above equation into (15), we have:

(A6) $${\mathrm{ADOP}}_{\mathrm{DAC}}={\sqrt{\left|\left[\begin{array}{cc} Q_{\nabla \Delta N_{1}} & \\ & Q_{\nabla \Delta N_{2}} \end{array}\right]-\left[\begin{array}{c} Q_{b_{1}\nabla \Delta N_{1}}^{T}\\ Q_{b_{2}\nabla \Delta N_{2}}^{T} \end{array}\right]W_{\varepsilon }\left[\begin{array}{cc} Q_{b_{1}\nabla \Delta N_{1}}^{} & Q_{b_{2}\nabla \Delta N_{2}}^{} \end{array}\right]\right|}}^{\frac{1}{n+m}}$$

If $\left|W_{\varepsilon }\right|\neq 0$, according to Equation (A4),

(A7) $${\mathrm{ADOP}}_{\mathrm{DAC}}{=\mathrm{ADOP}}_{\mathrm{DANOC}}{\sqrt{\left|W_{\varepsilon }^{-1}-\left[\begin{array}{cc} Q_{b_{1}\nabla \Delta N_{1}}^{} & Q_{b_{2}\nabla \Delta N_{2}}^{} \end{array}\right]\left[\begin{array}{cc} Q_{\nabla \Delta N_{1}}^{-1} & \\ & Q_{\nabla \Delta N_{2}}^{-1} \end{array}\right]\left[\begin{array}{c} Q_{b_{1}\nabla \Delta N_{1}}^{T}\\ Q_{b_{2}\nabla \Delta N_{2}}^{T} \end{array}\right]\right|}}^{\frac{1}{n+m}}$$

For DAVEC-based dual-antenna combination, since $W_{\varepsilon ,BV}={\left(Q_{b_{1}}+Q_{b_{2}}+R_{\varepsilon ,BV}^{}\right)}^{-1}$ is reversible, the ADOP can be rewritten as:

(A8) $$\begin{array}{l} {\mathrm{ADOP}}_{\mathrm{DAVEC}}=\\ {\mathrm{ADOP}}_{\mathrm{DANOC}}{\sqrt{\frac{\left|\left(Q_{b_{1}}-Q_{b_{1}\nabla \Delta N_{1}}^{}Q_{\nabla \Delta N_{1}}^{-1}Q_{b_{1}\nabla \Delta N_{1}}^{T}\right)+\left(Q_{b_{2}}-Q_{b_{2}\nabla \Delta N_{2}}^{}Q_{\nabla \Delta N_{2}}^{-1}Q_{b_{2}\nabla \Delta N_{2}}^{T}\right)+R_{\varepsilon }^{}\right|}{\left|Q_{b_{1}}+Q_{b_{2}}+R_{\varepsilon }^{}\right|}}}^{\frac{1}{n+m}} \end{array}$$

## Appendix B

The derivation of the DAVEC algorithm is given below.

### Appendix B.1. Measurement Projection Based on the Known Baseline Vector:

Proof:

(A9) $$\begin{array}{l} \lambda \nabla \Delta {\tilde{\Phi }}_{rb_{1}}^{ij}=\lambda \nabla \Delta \Phi_{r_{2}b}^{ij}-\nabla \Delta \rho_{r_{2}r_{1}}^{ij}-\lambda \nabla \Delta N_{r_{2}r_{1}}^{ij}=\nabla \Delta \rho_{r_{1}b}^{ij}+\lambda \nabla \Delta N_{r_{1}b}^{ij}+\nabla \Delta \varepsilon_{r_{2}b,\Phi }^{ij}\\ \nabla \Delta {\tilde{P}}_{r_{1}b}^{ij}=\nabla \Delta P_{r_{2}b}^{ij}-\nabla \Delta \rho_{r_{2}r_{1}}^{ij}=\nabla \Delta \rho_{r_{1}b}^{ij}+\nabla \Delta \varepsilon_{r_{2}b,P}^{ij} \end{array}$$

According to ([23], p. 170):

(A10) $$\begin{array}{l} \nabla \Delta \rho_{r_{1}b}^{ij}=-(1_{b}^{\left(i\right)}-1_{b}^{\left(j\right)})\cdot b_{r_{1}b}^{}\\ \nabla \Delta \rho_{r_{2}b}^{ij}=-(1_{b}^{\left(i\right)}-1_{b}^{\left(j\right)})\cdot b_{r_{2}b}^{} \end{array}$$

Then by subtracting $\nabla \Delta \rho_{r_{1}b}^{ij}$ from $\nabla \Delta \rho_{r_{2}b}^{ij}$, we have:

(A11) $$\begin{array}{cc} \nabla \Delta \rho_{r_{2}b}^{ij}-\nabla \Delta \rho_{r_{1}b}^{ij} & =-(1_{b}^{\left(i\right)}-1_{b}^{\left(j\right)})\cdot (b_{r_{2}b}^{}-b_{r_{1}b}^{})\\ & =-(1_{b}^{\left(i\right)}-1_{b}^{\left(j\right)})\cdot b_{r_{2}r_{1}}^{}=\nabla \Delta \rho_{r_{2}r_{1}}^{ij} \end{array}$$

Therefore,

(A12) $$\nabla \Delta P_{r_{2}b}^{ij}-\nabla \Delta \rho_{r_{2}r_{1}}^{ij}=\nabla \Delta P_{r_{2}b}^{ij}+\nabla \Delta \rho_{r_{1}b}^{ij}-\nabla \Delta \rho_{r_{2}b}^{ij}=\nabla \Delta \rho_{r_{1}b}^{ij}+\nabla \Delta \varepsilon_{r_{2}b,P}^{ij}$$

For the second equation in (A9), subtract $\nabla \Delta \Phi_{r_{1}b}^{ij}$ from $\nabla \Delta \Phi_{r_{2}r_{1}}^{ij}$:

(A13) $$\begin{array}{cc} \lambda \nabla \Delta \Phi_{r_{2}b}^{ij} & =\lambda \nabla \Delta \Phi_{r_{2}r_{1}}^{ij}-\lambda \nabla \Delta \Phi_{r_{1}b}^{ij}\\ & =\nabla \Delta \rho_{r_{2}r_{1}}^{ij}+\lambda \nabla \Delta N_{r_{2}r_{1}}^{ij}-\nabla \Delta \rho_{r_{1}b}^{ij}-\lambda \nabla \Delta N_{r_{1}b}^{ij}+\nabla \Delta \varepsilon_{r_{2}b,\Phi }^{ij} \end{array}$$

We have:

(A14) $$\lambda \nabla \Delta \Phi_{r_{2}b}^{ij}-\nabla \Delta \rho_{r_{2}r_{1}}^{ij}-\lambda \nabla \Delta N_{r_{2}r_{1}}^{ij}=\nabla \Delta \rho_{r_{1}b}^{ij}+\lambda \nabla \Delta N_{r_{1}b}^{ij}+\nabla \Delta \varepsilon_{r_{2}b,\Phi }^{ij}$$

End of proof.

### Appendix B.2. Noise Projection Based on the Known Baseline Vector:

According to ([23], p. 168), the SD geometric distance between r1 and r2 can be expressed as:

(A15) $$\Delta \rho_{r_{2}r_{1}}^{i}=-1_{r_{1}}^{\left(i\right)}\cdot C_{n}^{e}\cdot b_{r_{2}r_{1}}^{n}\approx -1_{b}^{\left(i\right)}\cdot C_{n}^{e}\cdot b_{r_{2}r_{1}}^{n}=1_{b}^{\left(i\right)}\cdot C_{n}^{e}\cdot \left[\begin{array}{c} e\\ n\\ u \end{array}\right]$$

where $C_{n}^{e}$ is the rotation matrix from ECEF to ENU.

The SD geometric distance $\Delta \rho_{r_{2}r_{1}}^{}$is derived as:

(A16) $$\Delta \rho_{r_{2}r_{1}}^{}=\left[\begin{array}{c} \Delta \rho_{r_{2}r_{1}}^{1}\\ \Delta \rho_{r_{2}r_{1}}^{2}\\ \vdots \\ \Delta \rho_{r_{2}r_{1}}^{N} \end{array}\right]=\left[\begin{array}{c} -{\left(1_{b}^{\left(1\right)}\right)}^{T}\\ -{\left(1_{b}^{\left(2\right)}\right)}^{T}\\ \vdots \\ -{\left(1_{b}^{\left(N\right)}\right)}^{T} \end{array}\right]\cdot C_{n}^{e}\cdot b_{r_{2}r_{1}}^{n}=\Theta^{T}C_{n}^{e}\cdot b_{r_{2}r_{1}}^{n}$$

The measurement noise in $\Delta \rho_{r_{2}r_{1}}^{}$ is expressed by:

(A17) $$\delta_{\Delta \rho_{r_{2}r_{1}}^{}}=\Theta^{T}\cdot C_{n}^{e}\left[\begin{array}{c} \delta e\\ \delta n\\ \delta u \end{array}\right]$$

Thus, the STD of $\delta_{\Delta \rho_{r_{2}r_{1}}^{}}$ can be estimated with:

(A18) $$\mathrm{var}\left(\delta_{\Delta \rho_{r_{2}r_{1}}^{}}\right)=E\left(\left(\delta_{\Delta \rho_{r_{2}r_{1}}^{}}-E\left(\delta_{\Delta \rho_{r_{2}r_{1}}^{}}\right)\right)\cdot {\left(\delta_{\Delta \rho_{r_{2}r_{1}}^{}}-E\left(\delta_{\Delta \rho_{r_{2}r_{1}}^{}}\right)\right)}_{}^{T}\right)$$

Therefore, the covariance matrix of SD baseline vector measurement is:

(A19) $$\begin{array}{cc} R_{\rho } & =\Theta^{T}\cdot C_{n}^{e}\cdot E\left(\left[\begin{array}{c} \delta e\\ \delta n\\ \delta u \end{array}\right]\cdot \left[\begin{array}{ccc} \delta e & \delta n & \delta u \end{array}\right]\right)\cdot {\left(C_{n}^{e}\right)}^{T}\Theta \\ & =\Theta^{T}\cdot C_{n}^{e}\left[\begin{array}{ccc} \sigma_{e}^{2} & 0 & 0\\ 0 & \sigma_{n}^{2} & 0\\ 0 & 0 & \sigma_{u}^{2} \end{array}\right]{\left(C_{n}^{e}\right)}^{T}\Theta \end{array}$$

Accordingly, for SD measurements of r2:

(A20) $$\begin{array}{l} \lambda \Delta {\tilde{\Phi }}_{rb_{1}}^{ij}=\lambda \Delta \Phi_{r_{2}b}^{ij}-\Delta \rho_{r_{2}r_{1}}^{ij}-\lambda \Delta N_{r_{2}r_{1}}^{ij}\\ \Delta {\tilde{P}}_{r_{1}b}^{ij}=\Delta P_{r_{2}b}^{ij}-\Delta \rho_{r_{2}r_{1}}^{ij} \end{array}$$

The projected carrier phase SD measurement error is obtained through:

(A21) $$\Sigma_{2}=\Sigma_{1}+R_{\rho }^{}$$

The projected pseudorange SD measurement error after projection:

(A22) $$\Sigma_{2}=\Sigma_{1}\times LPratio^{2}+R_{\rho }^{}$$
